# The Study of the Acoustic Characteristics of Chitosan Acetate Film Using a Radial Electric Field Excited Resonator

**DOI:** 10.3390/s23041808

**Published:** 2023-02-06

**Authors:** Andrey Teplykh, Boris Zaitsev, Alexander Semyonov, Irina Borodina

**Affiliations:** Kotel’nikov Institute of Radio Engineering and Electronics of RAS, Saratov Branch, 410019 Saratov, Russia

**Keywords:** acoustic resonator, circular piezoceramic disk, finite element method, electrical impedance, acoustic resonance spectroscopy, chitosan acetate film, ammonia, Nelder-Mead algorithm

## Abstract

Currently, the lateral electric field excited resonators are used for the creation of various sensors. We have recently proposed a new type of acoustic resonator called radial electric field excited disk acoustic resonator. The advantage of this type of resonator is its high sensitivity to mechanical and electrical boundary conditions on its free surface. This makes it possible to determine both the acoustic and electrical properties of a thin layer of material deposited on the free end of the resonator. In this work, we used a radial electric field excited disk acoustic resonator of Russian-made barium plumbum zirconate titanate (BPZT) piezoceramics. With the help of this resonator, the material constants for the piezoceramic sample were refined, and their temperature dependencies were determined. Then, this resonator was used to determine the elastic modulus, viscosity, and conductivity of the chitosan acetate film in air and ammonia vapors of various concentrations. It was shown that the chitosan acetate film under the influence of ammonia vapor significantly changes its mechanical properties and increases its electrical conductivity thousands of times, and then completely restores its properties.

## 1. Introduction

Thin films and structures based on them have a number of unique physical and chemical properties that differ from those of bulk materials [1,2]. This has made them a relevant object of experimental and theoretical research for more than a decade. It is also known that the mechanical and electrical properties of films can be determined using acoustic methods. For example, to determine the elastic modulus, viscosity coefficient, and density of thin films, piezoelectric resonators with a longitudinal electric field can be used [3,4,5,6,7,8]. This resonator represented a piezoelectric disk with electrodes on each side, and the film under study was deposited on one of these electrodes. The frequency dependencies of the electrical impedance of a free resonator and one loaded with a film were measured. Then, by fitting the theoretical frequency dependencies of the impedance to the experimental ones, the modulus of elasticity, the viscosity coefficient, and the density of thin films were determined. This method was used to study the mechanical properties of silicon dioxide films [3], nanocomposite films based on low-pressure polyethylene [4], mycelial films [5,6,7], and chitosan films [8,9] in the presence of various gases. There is also an approach in which surface acoustic waves are used to determine the mechanical properties of films [10]. The longitudinal and shear moduli of elasticity, as well as the density of silicon dioxide films, were determined in this work. However, the listed approaches show the possibility of determining only the mechanical properties of the film and do not allow evaluation of their electrical properties.

In order for acoustic methods to determine not only the mechanical but also the electrical properties of the layer, the electric field that accompanies acoustic oscillations in a piezoelectric material must freely penetrate into the layer. This means that the medium must be in direct contact with the resonator material, and not with the metal electrode on its surface. In this regard, lateral electric field excited piezoelectric resonators are very promising [11,12,13,14,15,16]. Resonators of this type are sensitive not only to the mechanical but also to the electrical properties of the medium that borders on the free surface of the resonator [8,9,17]. For example, the effect of an electrically conductive layer located near the free side of a resonator on its characteristics has been studied in [11,12]. This opens up the possibility of non-contact determination of the conductivity of thin films. Obviously, the mechanical properties of the films cannot be determined in this case. It is also known that the conductivity of films can be determined using a delay line with a shear horizontal acoustic wave propagating in a lithium niobate plate [18].

Thus, to date, no approaches have demonstrated the possibility of simultaneously determining the mechanical and electrical properties of thin films.

In this work, we propose using the method of broadband acoustic resonance spectroscopy (ARS) [8,9] to directly calculate the mechanical and electrical properties of the films under study. This work should be done in two stages. First, the electrical impedance is measured in a fairly wide frequency range, which allows us to find the resonant frequencies for a free piezoelectric resonator. At this stage, a finite element model of the resonator is built, and the characteristics of the material (elastic constants, density, viscosity) of the resonator itself can be refined. This is accomplished through the use of broadband acoustic resonance spectroscopy, as shown in [8,9,17,19]. Then, a layer of the material under study is deposited to the free surface of the resonator, and the measurements are repeated in the same frequency range. By changing the values of resonant frequencies and the magnitude of the resonant peaks, one can judge the properties of the material under study [7,8,9].

To apply the method of broadband acoustic resonant spectroscopy, one should easily be able to computationally determine the resonator oscillation spectrum (the natural frequencies) or the resonator response (the electrical impedance) to the excitation at a certain frequency [20]. Our studies have shown the possibility of accurately and quickly calculating the oscillations and electrical impedance of a piezoelectric disk made of a piezomaterial of the 6 mm group, the crystallographic axis of which coincides with the axis of the disk. In this case, one can strictly take into account the different positions of the exciting electrodes and the inhomogeneity of the disk material if this does not violate the axial symmetry of the problem [21]. This allows us to determine the acoustic properties of the substance layer deposited on the free side of the resonator. Previously, a similar problem was solved for a circular resonator with a longitudinal excitation field [7,8,9].

In this paper, we propose the design of such a resonator in the form of a round piezoceramic disk with a radial excitation electric field loaded with the film under study. A mathematical model of such a loaded resonator was created based on the finite element method. Theoretical and experimental results were compared for Russian-made barium plumbum zirconate titanate (BPZT) piezoceramics. The material constants of piezoceramics were refined, and their linear temperature dependencies near the room temperature were determined. As a result of this work, a disk acoustic resonator with a radial excitation field was used to determine the moduli of elasticity, viscosity, permittivity, and electrical conductivity of a chitosan acetate film.

## 2. Materials and Methods

### 2.1. Numerical Model of a Free Radial Electric Field Excited Circular Piezoresonator

We solve the problem of forced oscillations of a circular piezoceramic disk excited by a pair of concentric electrodes located on one side of the disk. Such a problem was considered in [22], so here we will only briefly recall its formulation. A disk with diameter *d* and thickness *h*, made of piezoceramics belonging to the crystallographic class of 6 mm, is considered. Let the axis of polarization of the ceramics be parallel to the axis of disk *z*. The concentric metal electrodes 1 and 2 are located on the underside of the disk. Electrode 1 has a radius *e*_1_, its center coincides with the center of the disk, and the gap between the electrodes is *g*. Electrode 2 has the shape of a ring with an inner radius *e*_2_ and extends to the outer edge of the disk; thus, *e*_1_ + *g* = *e*_2_. The side surface and the upper side of the disk are mechanically and electrically free. The upper side of the disk can also be free or bordered with the viscoelastic film under study. Due to the absence of metal surfaces on the upper side of the disk, the electric field freely penetrates into the space above the disk.

The mathematical problem is to determine the components of the acoustic field and the electric potential inside the disk. This problem is axisymmetric since the axis of the disk is parallel to the direction of polarization of the piezoceramic. Obviously, it can be written in a two-dimensional form with respect to the cylindrical coordinates *r* and *z*. The solution to the problem should be written in the following form:

(1) $$\begin{array}{r} u_{r}=u_{r}\left(r,z\right)\exp\left(I\omega t\right)\\ u_{z}=u_{z}\left(r,z\right)\exp\left(I\omega t\right)\\ \varphi =\varphi \left(r,z\right)\exp\left(I\omega t\right) \end{array}\},$$

Here, *u_r_* and *u_z_* are, respectively, the radial and axial components of mechanical displacement, *φ* is the electrical potential, *I* is the imaginary unit, *ω* is the angular frequency, and *t* is the time. Since there is no mechanical displacement around the disk axis in this formulation, the variable *u_θ_* can be completely omitted from the equations.

As shown in [23], we may take into account only four components of the deformation *S*,

(2) $$\left\{S\right\}=\left\{\begin{array}{c} S_{rr}\\ S_{\theta \theta }\\ S_{zz}\\ 2S_{rz} \end{array}\right\}=\left[\begin{array}{cc} \frac{\partial }{\partial r} & 0\\ \frac{1}{r} & 0\\ 0 & \frac{\partial }{\partial z}\\ \frac{\partial }{\partial z} & \frac{\partial }{\partial r} \end{array}\right]\left\{\begin{array}{c} u_{r}\\ u_{z} \end{array}\right\}=\left[L_{u}\right]\left\{u\right\},$$

and two components of the electrical field *E*.

(3) $$\left\{E\right\}=\left\{\begin{array}{c} E_{r}\\ E_{z} \end{array}\right\}=-\left[\begin{array}{c} \frac{\partial }{\partial r}\\ \frac{\partial }{\partial z} \end{array}\right]\varphi =-\left[L_{\varphi }\right]\varphi$$

In the two-dimensional axisymmetric case under consideration, some rows and columns can be removed from the material constant tensors, as shown in [21], so these tensors can be written in the following matrix form:

(4) $$\left[c\right]=\left[\begin{array}{cccc} c_{11} & c_{12} & c_{13} & 0\\ c_{12} & c_{11} & c_{13} & 0\\ c_{13} & c_{13} & c_{33} & 0\\ 0 & 0 & 0 & c_{44} \end{array}\right]\left(1+I\omega \eta \right),\;\left[e\right]=\left[\begin{array}{cccc} 0 & 0 & 0 & e_{15}\\ e_{31} & e_{31} & e_{33} & 0 \end{array}\right],\;\left[\varepsilon \right]=\left[\begin{array}{cc} \varepsilon_{11} & 0\\ 0 & \varepsilon_{33} \end{array}\right].$$

It follows from (4) that the resonator material is fully characterized by five elastic constants *c*_11_, *c*_12_, *c*_13_, *c*_33_, *c*_44_, three piezoelectric constants *e*_15_, *e*_31_, *e*_33_, two dielectric constants *ε*_11_ and *ε*_33_, density *ρ*, and scalar viscosity factor *η*.

Thus, in the theoretical analysis of the characteristics of the resonator, it was assumed that the losses are determined only by the presence of friction, which is determined by the viscosity coefficient. Other sources of the losses, such as thermoelastic [23] and electrical [24] losses, were not taken into account due to the low values of the thermal and electrical conductivities of the material.

The corresponding boundary conditions are also considered [22]. A mechanical boundary condition is specified on the disk axis in the following form:

(5) $${u_{r}=0|}_{r=0}.$$

The electrical boundary conditions on the underside of the disk are as follows:

(6) $${\varphi =+V|}_{0\leq r\leq e_{1}},\;{\varphi =-V|}_{e_{2}\leq r\leq d}$$

where *V* is the RF electric voltage applied to the electrodes with angular frequency *ω*. The side surface and the other end of the disk are mechanically and electrically free.

To solve this problem, we use the axially symmetric 2D finite element method. This allows us to calculate the electrical impedance between the resonator electrodes for a given frequency, taking into account the known material constants of piezoceramics and the geometry of the resonator (this is a solution to a direct problem). In turn, the solution to the inverse problem allows us to find the refined material components of the piezoceramics using the measured electrical impedance curve in the sufficiently wide frequency range.

### 2.2. Numerical Model of a Radial Electric Field Excited Circular Piezoresonator Loaded with a Viscoelastic Film, Which Has a Finite Thickness and a Finite Electrical Conductivity

The above-described mathematical model of a circular resonator with a radial exciting electric field can be supplemented with a layer of viscoelastic material of finite thickness with finite electrical conductivity, which is located on the free side of the piezodisk [25].

Now, we suppose that a plane-parallel layer of isotropic viscoelastic material with thickness *f* is located on the upper side of the disk. This material is characterized by two material elasticity modules *c^f^*_11_ and *c^f^*_44_, viscosity coefficients *η^f^*_11_ and *η^f^*_44_, permittivity *ε^f^*, electrical conductivity *σ^f^*, and density *p^f^*. The corresponding effective tensors can be written as:

(7) $$\left[c^{f}\right]=\left[\begin{array}{cccc} c_{11}^{f} & c_{12}^{f} & c_{12}^{f} & 0\\ c_{12}^{f} & c_{11}^{f} & c_{12}^{f} & 0\\ c_{12}^{f} & c_{12}^{f} & c_{11}^{f} & 0\\ 0 & 0 & 0 & c_{44}^{f} \end{array}\right],\;\left[\eta^{f}\right]=\left[\begin{array}{cccc} \eta_{11}^{f} & \eta_{12}^{f} & \eta_{12}^{f} & 0\\ \eta_{12}^{f} & \eta_{11}^{f} & \eta_{12}^{f} & 0\\ \eta_{12}^{f} & \eta_{12}^{f} & \eta_{11}^{f} & 0\\ 0 & 0 & 0 & \eta_{44}^{f} \end{array}\right],\;\left[\varepsilon^{f}\right]=\left[\begin{array}{cc} \varepsilon^{f}-\frac{I\sigma^{f}}{\omega } & 0\\ 0 & \varepsilon^{f}-\frac{I\sigma^{f}}{\omega } \end{array}\right]$$

where *c^f^*_12_ = *c^f^*_11_ − 2*c^f^*_44_, *η^f^*_12_ = *η^f^*_11_ − 2*η^f^*_44_.

The distributions of the corresponding fields inside the viscoelastic layer and the piezoelectric are crosslinked using the boundary conditions of continuity:

(8) $${\begin{array}{l} u_{i}-u_{i}^{f}=0,\\ \left(T_{ij}-T_{ij}^{f}\right)n_{j}=0,\\ \varphi -\varphi^{f}=0,\\ \left(D_{j}-D_{j}^{f}\right)n_{j}=-\delta \end{array}|}_{z=h/2}$$

where the values with the upper index *f* refer to the film, *n_j_* is the component of the boundary normal, *T* is the stress tensor, *D* is the electric induction, and *δ* is the density of the electric charge in the film. In this work, we assumed that *δ* = 0.

As shown in [25], the solution of this problem by the finite element method allows us to calculate the electrical impedance of a film-coated disk for a specified frequency *ω* using the known constants of the piezodisk and film. The solution of the inverse problem for this case, using the preliminarily refined material constants of the piezoceramic of the disk, allows us to determine the acoustic parameters of the material of the film covering the upper side of the disk, as well as its electrical conductivity.

### 2.3. Features of the Finite Element Resonator Model Containing a Thin Film

**Figure 1:**
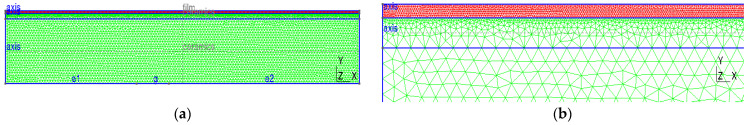
Finite element model of a resonator loaded with a viscoelastic conducting layer: (**a**) general view of the model; (**b**) enlarged view near the layer. Blue lines are the boundaries of the model, green the resonator, and red the layer.

To determine the maximum size of the finite element, which is necessary to perform calculations with acceptable accuracy, we use the following simple estimate. The velocity of the shear acoustic wave in the studied piezoceramics is *v_s_* = 1950 m/s. In addition, the velocity of the shear acoustic wave in the film under study, according to our estimates, is approximately *v^f^_s_* = 2000 m/s. At the frequency *f* = 2 MHz, the length of the shear acoustic wave is *λ_s_* = 1 mm. In accordance with the recommendations from [26], the maximum transverse size of the finite element for a FEM model capable of correctly describing such oscillations should be no more than 0.1 mm (100 μm). However, the average thickness of the film under consideration is 60 μm, while at least 5 elements should fit in the thickness, so the size of element *a* in the film should not exceed 12 μm. For the rest of the model, this is redundant. Therefore, for the correct description of oscillations in a resonator loaded with a film, a model consisting of three layers was used. The main layer describing the thickness of the ceramic resonator, *a* = 80 microns, the size of the element in the transition layer in ceramics near the surface of the resonator changes smoothly in the range of *a* = 80.12 microns, while the material constant remains unchanged. The size of the element in a layer of viscoelastic conductive film is *a* = 12 microns. This model is shown in Figure 1.

This model was created using the open-source software Gmsh 4.8.4 [27]. It contains 13,465 nodes and 26,922 linear elements, which is 3.28 times more than a similar model of a free resonator without a transition layer.

### 2.4. Creation of a Disk Resonator with a Radial Exciting Field from BPZT Piezoceramics

In our previous works [22,25], the resonators were made from blanks purchased from Aurora-ELMA LLC, Volgograd, Russia. The commercial name of this ceramic is VA-650, and its chemical composition is Pb_0.75_Ba_0.25_(Zr_0.53_Ti_0.47_)O_3_. As was shown in [22], this ceramic has the required symmetry, and its resonators are characterized by a high quality factor (Q > 2400 for the F_0_ mode) and a high electromechanical coupling coefficient (K^2^ = 6.6% for the F_6_ mode). So, this material is the most suitable for our purpose among all Russian-made ceramics available to us. At that time, thin gold wires were used to create an electrical contact, which was glued to the surface of the electrodes using a special conductive glue. As it turned out, this glue does not have resistance to the atmosphere of ammonia, so the technology of creating a resonator needs to be changed. Preliminary experiments have shown that it is impossible to solder or weld a thin gold wire to a very thin aluminum electrode. Therefore, for this purpose, we decided to keep part of the factory-deposited silver electrodes with a thickness of about 20 μm. For this, two drops of the acid-resistant varnish were placed on the low side of the resonator: in the center and near the edge. The rest of the surface and the second side were left free. After the varnish dried, the resonator was immersed in a solution of nitric acid. All silver electrodes, with the exception of the two protected areas, were dissolved and removed. Then, the resonator was washed with distilled water, and the varnish drops were removed with alcohol. As a result, two silver islands with a diameter of about 3 mm remained on one side of the resonator, and the rest of the surface was cleaned of silver, while the surface layer of ceramics remained intact.

After removing the electrodes, the diameter, thickness, and mass of the resonator blank were measured. The diameter of the resonator was measured with a caliper, and the thickness was measured with a micrometer probe by averaging the values of 5 measurements at different points. The resonator mass was determined using OHAUS electronic balances.

**Figure 2:**
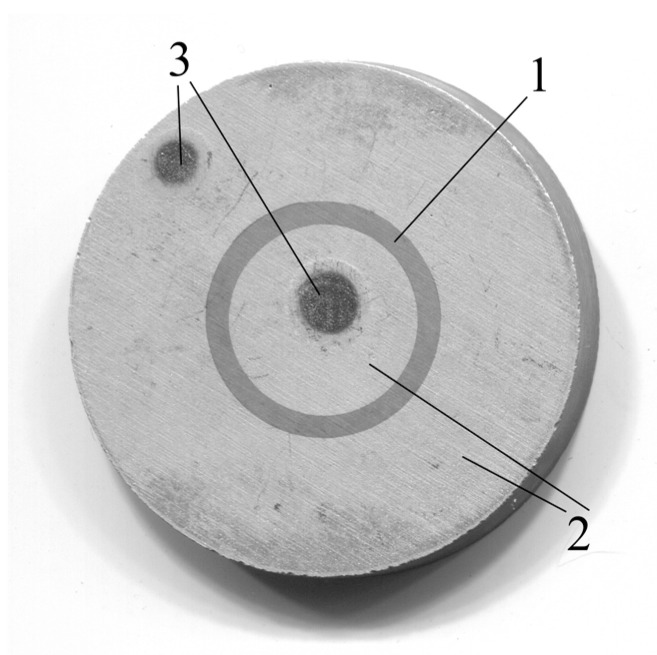
Photo of a BPZT piezoceramic radial electric field excited resonator. 1—piezoceramics, 2—aluminum electrodes, and 3—areas of soldering wires.

Then, new aluminum electrodes were applied to the piezoceramic blank. The application of new aluminum electrodes was carried out by vacuum spraying on the VUP 5 installation through a specially made nickel mask in the form of a ring. The ring was fixed on top of the piezoceramic disk using a permanent magnet. The position of the mask on top of the resonator was controlled using a microscope, so the positioning accuracy was about 20 μm. The thickness of the obtained aluminum electrodes was about 2000 Å. The resulting resonator is shown in Figure 2.

The size and position of the resulting electrodes were monitored using a microscope. As a result, a resonator was manufactured with the characteristics shown in Table 1.

**Figure 3:**
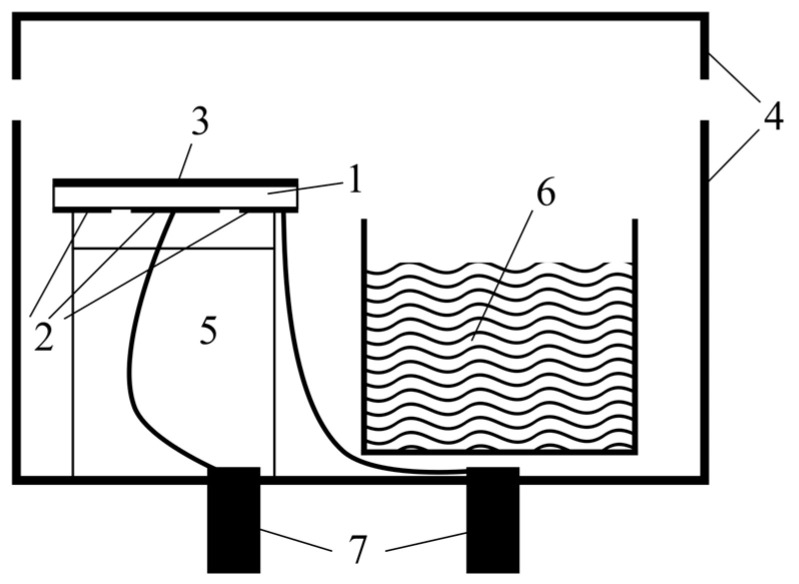
Gas chamber side view: 1—piezoresonator, 2—electrodes, 3—chitosan acetate film, 4—camera body and sealing cover, 5—solid support with top low impedance support, 6—volatile liquid (water ammonia solution) in container, and 7—electrodes for the impedance analyzer port.

The pieces of a copper wire with a diameter of 25 μm and a length of 30 mm were soldered to the silver islands at the side of the resonator. With the help of this wire, the resonator was soldered to the impedance analyzer port electrodes, and the resonator itself was placed with the electrodes down on a special holder made of the low impedance foam rubber, which was placed inside a 100 mL plastic container with a hermetically sealed lid (Figure 3).

### 2.5. Measurement of Resonator Characteristics. Taking into Account the Influence of Temperature

It is known that piezoceramics based on PZT have low temperature stability [28], i.e., their material constants change greatly with the temperature changes, which leads to a shift in the resonant frequencies of the resonator. Therefore, the first goal of the work was to determine the values of these material constants at different temperatures and to construct temperature dependencies for each constant.

**Figure 4:**
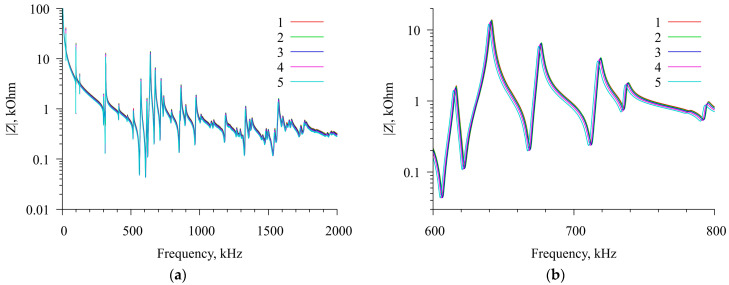
Frequency dependencies of the module of the electrical impedance of a free radial electric field excited resonator at different temperatures. (**a**) General view of dependencies; (**b**) magnified view in the range of 600–800 kHz. Curves 1—25 °C, 2—30 °C, 3—35 °C, 4—40 °C, and 5—45 °C.

For this, a plastic chamber with a resonator was placed in a thermostat, which could change and maintain a constant temperature with an accuracy of 0.1 °C for an arbitrary long time. The relative humidity of the air inside the thermostat did not exceed 20%. After reaching the required temperature, the real and imaginary components of the electrical impedance of a resonator with a radial field were measured. All measurements were performed using the impedance analyzer E4990A (Keysight Technologies) after proper pre-calibration. The measurements were carried out in the frequency range of 1 kHz–2001 kHz in increments of 10 Hz. These measurements were carried out several times at temperatures from 25 °C to 45 °C in increments of 5 °C. During these experiments, the camera lid was open. The impedance measurement results for the free resonator are shown in Figure 4.

### 2.6. Creation of a Chitosan Acetate Film on the Resonator Surface

In this study, we used chitosan acetate because it is one of the most widely studies water-soluble derivatives. We plan to measure the change in the mechanical parameters of chitosan films (longitudinal and shear elastic modulus and viscosity coefficient) in the presence of ammonia vapor. Therefore, the chitosan film applied to the surface of the piezoresonator was subject to such requirements as the plane parallelism of the sides and the absence of surface roughness. It also has the simplest solution preparation protocol among chitosan organic acid salts, which provide a smoother surface compared to inorganic acid salts. Chitosan acetate was obtained by heterogeneous synthesis, i.e., briefly, chitosan (LLC “Bioprogress,” Moscow, Russia) with a molecular weight of 150–200 kDa was added to a solution of acetic acid (Sigma Aldrich, St. Louis, MO, USA) in an aqueous-ethanol mixture. The mixture was re-stirred for 3 h at 50 °C. The resulting precipitate was filtered and dried on a rotary evaporator at a residual pressure of 15 mbar and a temperature of 50 °C. We prepared a 1.5% aqueous solution using the resulting chitosan acetate. Then, this solution was poured onto the horizontal surface of the resonator, free of electrodes, with a layer 2 mm thick. On the sides, the resonator was limited by a special temporary border made of scotch tape. After complete drying of the solution at room temperature for 72 h, a sufficiently smooth and homogeneous layer of chitosan acetate was formed on the surface of the resonator, and then the tape was removed.

Measuring the thickness with a micrometer probe and weighing the resonator with a chitosan acetate film showed that the film has a thickness of 60–70 μm and the density of the dry chitosan acetate film calculated on the basis of mass and volume was *ρ^f^* = 700 kg/m^3^. This is in satisfactory agreement with the results from [8].

### 2.7. Measurement of the Characteristics of a Resonator Loaded with a Chitosan Acetate Film in an Ammonia Atmosphere

**Figure 5:**
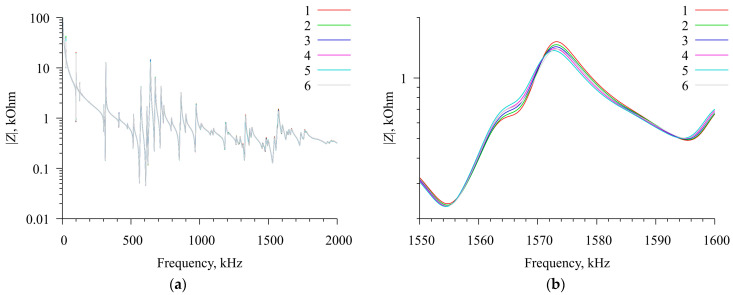
Frequency dependencies of the electrical impedance modulus for a radial electric field excited resonator loaded with a film of chitosan acetate at different times of exposure to ammonia vapors. (**a**) General view of dependencies; (**b**) magnified view in the range of 1550–1600 kHz. Curves 1—0 min, 2—100 min, 3—200 min, 4—300 min, 5—400 min, and 6—480 min from the beginning of the experiment.

The characteristics of a radial electric field excited resonator loaded with a film of chitosan acetate in ammonia were measured as follows. In this series of measurements with the resonator inside the chamber, the thermostat was not used. In this case, the temperature in the room was continuously measured by an electronic thermometer. The camera with the resonator and the film was connected to the measuring port of the impedance analyzer E4990A, and a series of measurements were carried out with the camera lid open in the pointed above frequency range. Then, a container with a 10% aqueous solution of ammonia with a volume of 6 mL was placed inside the chamber and the chamber lid was closed. The free evaporation of ammonia into the air atmosphere of the chamber began. At the same time, there was a continuous automatic measurement and recording of the real and imaginary parts of the electrical impedance in the range of 1–2001 kHz; each measurement took about 120 s. These measurements were repeated for 7 h; then, the chamber lid was opened, the container with ammonia solution was removed, and the measurements continued for another 1 h. The temperature of the chamber during the entire measurement cycle varied in the range of 26–27 °C. As a result of this series of measurements, loaded resonator spectra were obtained at different concentrations of ammonia in the air, which caused changes in the mechanical and electrical properties of the film and led to a minor change in the resonant frequencies of the loaded resonator (Figure 5). The dependence of the concentration of ammonia in the air on time is discussed in detail in [8]. In the presented work, we did not set out to determine the dependence of the conductivity of the chitosan film on the concentration of ammonia in the air, but simply used this effect to smoothly change the conductivity of a thin layer on the resonator surface.

The next day (18 h after the end of the main experiment), a control measurement was carried out, which showed that the frequency dependencies completely restored their original appearance, i.e., the chitosan acetate film restored its properties.

## 3. Results

### 3.1. Measurement Results for a Free Resonator. Determination of Temperature Dependencies of Material Constants of BPZT Piezoceramics

**Figure 6:**
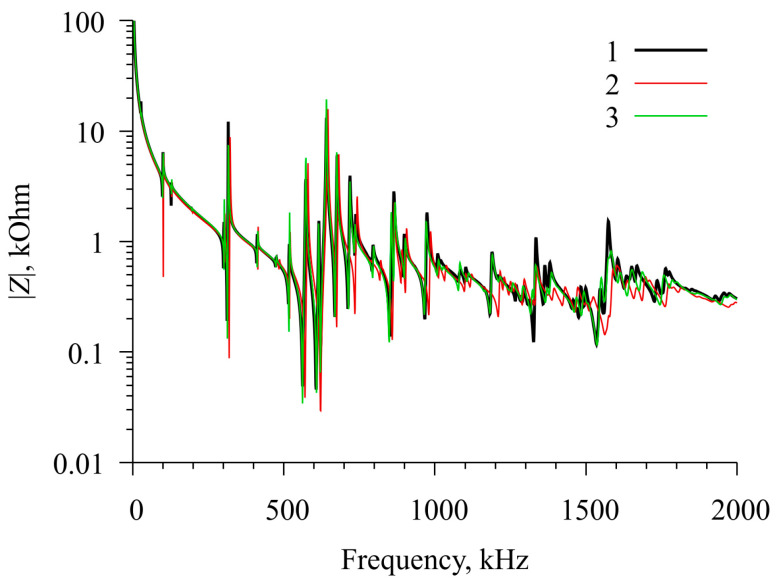
Comparison of the results of the measurement and calculation of the frequency dependence of the electrical impedance for a free radial electric field excited resonator at a temperature of 35 °C. Curve 1 is the measurement result, curve 2 is the result of calculation using the original material constants, and curve 3 is the result of calculation using the refined values of the material constants.

So, the method described in Section 2.1 allows the simulation of a free radial electric field excited piezoelectric resonator, the geometry of which, i.e., the diameter, thickness, and position of electrodes, exactly corresponds to the experimental sample. These characteristics are given in Table 1. This allows us to solve the so-called “direct problem,” i.e., to find the distribution of the acoustical and electrical fields inside the piezoresonator and its electrical impedance at a given frequency of the exciting field, taking into account the given material constants of piezoceramics. The results of the calculations are shown in Figure 6, curve 2. As one can see, this frequency dependence qualitatively coincides with the measurement results; however, the specific values of the calculated resonant frequencies differ markedly from the measurement results. This can be explained by the discrepancy between the material constants used in the calculation and the material constants of this particular sample of piezoceramics. Therefore, it is necessary to solve the “inverse problem” and refine the material constants of piezoceramics for a specific resonator.

**Figure 7:**
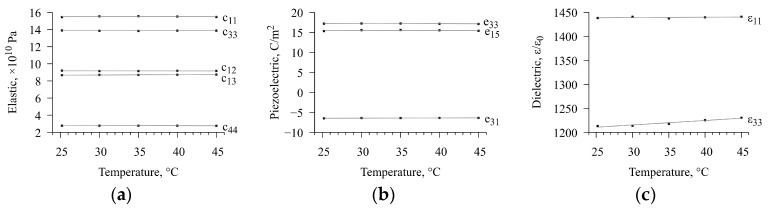
Dependencies of the material constants of piezoceramics on temperature. Dots are the results of fitting, and straight lines are the result of linear regression. (**a**) Elastic moduli; (**b**) piezoelectric moduli; (**c**) dielectric permittivity.

The parameters of the model that were subject to refinement were 10 material constants (5 independent components of the elastic moduli, 3 components of the piezomoduli, and 2 components of the dielectric permittivity). The procedure for refining the material constants (fitting) was carried out for each of the measured resonance curves at 5 different temperatures. The geometry of the resonator and the density of the ceramics were rigidly set and did not change during the fitting process. The Nelder-Mead algorithm [29] with adaptive parameters was used for fitting, which increased the convergence rate of the algorithm [30]. The completely fitting procedure is described in detail in [22,31,32]. The refined values for each material constant at a certain temperature are graphically represented in Figure 7 as black dots. Then, the values of material constants at a temperature of 35 °C and temperature coefficients with a temperature change of 1 °C were determined using the linear regression method, which is presented in Table 2. These results are presented in Figure 7 in the form of straight gray lines. The density of the ceramics was determined directly. The initial values of the remaining material constants were taken from [33].

The data obtained in this way allows to determine the optimal values of material constants for any sample temperature in the range of 25–45 °C. These results were used in the next stage of the work, when direct control of the temperature of the chamber with a resonator was impossible.

### 3.2. Measurement Results for a Resonator Loaded with a Chitosan Acetate Film. Determination of the Mechanical Characteristics and Conductivity of the Film at Different Concentrations of Ammonia

The main purpose of this work is to test the possibility of simultaneously determining the values of the acoustic and electrical characteristics of a thin viscoelastic layer with finite electrical conductivity deposited on the surface of a radial electric field excited resonator. As mentioned in Section 2.5, a chitosan acetate film with a thickness of about 60 microns was used as such a layer. As is known [8], this substance is capable of increasing its electrical conductivity many times in the presence of a certain amount of ammonia in the surrounding atmosphere. This property makes it very well suited for our task.

**Figure 8:**
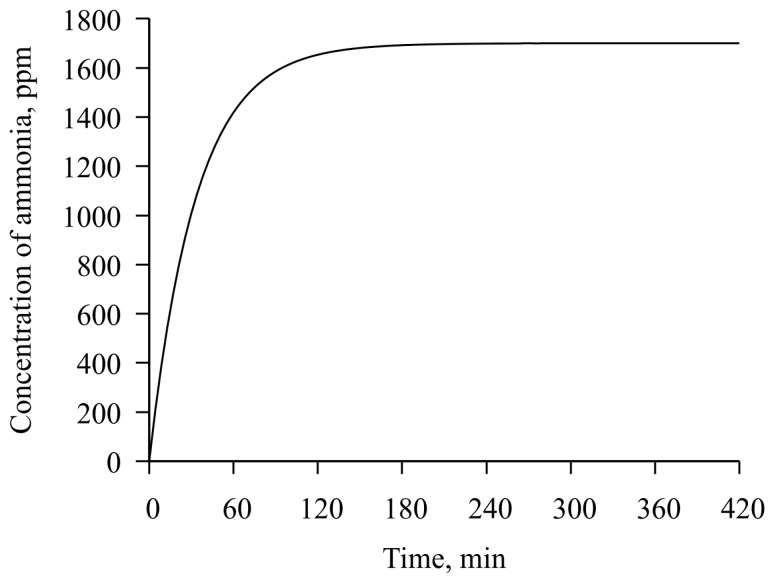
Computed time dependencies of ammonia concentration in air, by weight, in a gas chamber.

First, based on the free internal volume of the chamber (75 mL) and the area from which ammonia was evaporated from water solution (2.8 cm^2^), as well as the ambient temperature (26 °C), the expected concentration of ammonia in the air atmosphere for our chamber from time was calculated (Figure 8).

As follows from this dependence, within 120 min after closing the lid, an ammonia concentration of 1700 ppm is reached in the chamber, which remains practically constant until the lid is opened, after which it sharply decreases to 0 ppm. However, the rate of absorption of ammonia by the chitosan acetate film will have a more complex character, and in this experiment, it remained unknown because it is not the purpose of this study.

Then, the effective values of the material constants of the piezo ceramics of the resonator were determined for a temperature of 26 °C. This was done by linear interpolation of the values in Table 2. The obtained values were fixed and used unchanged for further calculations.

Then, an attempt was made to fit all the values of the material constants using the model from Section 2.3 describing a resonator loaded with a viscoelastic layer of finite thickness. In this model, the variable parameters were two elastic moduli *c^f^*_11_ and *c^f^*_44_, two viscosity moduli *η^f^*_11_ and *η^f^*_44_, dielectric permittivity *ε^f^*, and electrical conductivity *σ^f^*. The film thickness *f* and the density *ρ^f^* were determined before the experiment and were not the goals of fitting. However, it turned out that with such a formulation of the problem, it is not possible to determine the parameter of the dielectric permittivity of the film *ε^f^*. During repeated runs of the fitting algorithm with different initial conditions, it converged to different local minima, which differed in the parameter *ε^f^* by tens of times, i.e., the value of the relative permittivity *ε^f^* was in the range 1.50. This can be explained by the fact that the dielectric permittivity of piezoceramics exceeds 1000; therefore, the change in the dielectric permittivity of a thin film has practically no effect on the solution, i.e., the shape of the resonant curve. Therefore, during further runs of the algorithm, the value of *ε^f^* = 1.1 was fixed, as shown in [8]. The remaining parameters—elastic moduli, viscosity, and electrical conductivity—had a noticeable effect on the solution, so they could be determined with sufficient accuracy.

**Figure 9:**
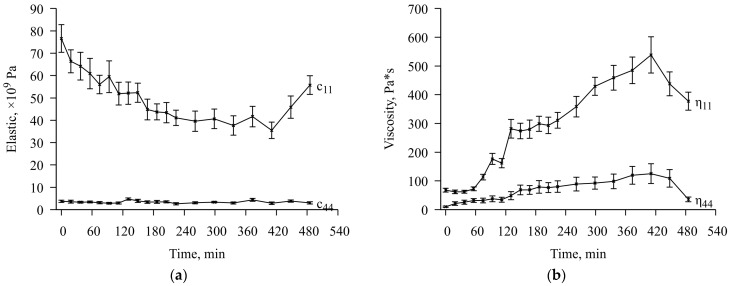
The results of fitting the mechanical material constants of the chitosan acetate film from the run time of the experiment. (**a**) elastic moduli; (**b**) viscosity moduli.

**Figure 10:**
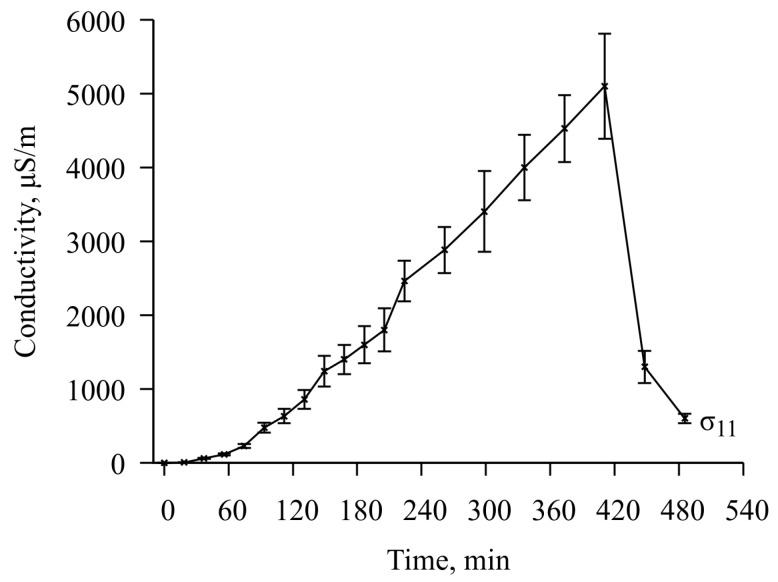
The results of fitting the values of the conductivity of the chitosan acetate film from the run time of the experiment.

The film thickness was determined with some error; therefore, after debugging the fitting procedure, launches were carried out with the film thickness parameters *f* = 60 μm and *f* = 70 μm, which led to two different sets of solutions. This allowed us to determine the error of the solution, which was about 10% for the elasticity and viscosity modules and about 15% for conductivity. The fitting results are shown in Figure 9 and Figure 10.

As a result, it was found that with the camera lid closed for 420 min, the elastic modulus of the chitosan acetate film decreases by about 50%, the viscosity increases by 6 times, and the electrical conductivity increases by 4250 times (the dry film conductivity is 1.2 µS/m., the maximum conductivity is 5100 µS/m). At the same time, despite the constant concentration of ammonia within 120–420 min after the start of the experiment, the conductivity value continues to increase almost uniformly. The growth limit of this parameter was not reached in this experiment. After opening the lid for 60 min, the values of all parameters decrease rapidly, but complete relaxation does not occur. The final restoration of the initial parameters of the chitosan acetate film takes no more than 18 h.

This is explained by the fact that in the presence of ammonia, the properties of the chitosan film change due to two processes: the adsorption of the ammonia molecules by the film and the change in its chemical composition. In air, adsorbed ammonia molecules leave the film relatively quickly, while chemical recovery turns out to be a longer process.

It should be noted that during the fitting procedure, the value of the film density and its thickness were assumed to be known and constant values. At the same time, during the experiment, after closing the camera lid, these parameters were not controlled. However, immediately after opening the lid, the surface of the chitosan acetate film looked swollen, i.e., its thickness was probably greater than at the beginning of the experiment. This effect was not taken into account during the calculations.

## 4. Discussion

As a result of this work, it was shown that using a radial electric field excited circular piezoelectric resonator and broadband acoustic resonance spectroscopy, it is possible to determine both the mechanical and electrical properties of thin films deposited on the free side of the resonator. This determines the novelty and advantages of the proposed method since previous approaches allowed us to determine either only the mechanical properties of the film or only the electrical ones. For the successful completion of this process, the film must be thick enough, and all the determined parameters of the film must influence the resonance curve for a loaded resonator, i.e., the position and magnitude of the resonant peaks should change when changing any defined parameter.

Using the method presented in the paper, the elastic moduli, viscosity moduli, and electrical conductivity of the chitosan acetate film were determined. For the first time, all these parameters were determined for one specific film sample during a single experiment. It is shown that in an ammonia atmosphere, the conductivity of the chitosan acetate film can increase by more than 3 orders of magnitude, and then all the parameters of the film return to their original values. These data qualitatively confirm the results obtained in [8].

In addition, as a result of this work, linear temperature dependencies of all material constants for Russian-made BPZT piezoceramics were determined for the first time. This allowed us to abandon the control of the sample temperature during experiments with a loaded resonator, which greatly simplified these experiments.

In general, the results obtained open up the possibility of developing a gas analyzer based on a radial electric field excited resonator and a chitosan acetate film sensitive to low concentrations of ammonia in the air.

## Figures and Tables

**Table 1:** Measured values of resonator properties.

Property	Value
Diameter (mm)	21.89
Thickness (μm)	1930
Mass (g)	5.287
Density (kg/m^3^)	7347
Radius *e*_1_	4.05
Radius *e*_2_	5.04

**Table 2:** Temperature dependencies of material constants for a sample of BPZT piezoceramics.

Constant	Reference Value [33]	Value at t = 35 °C	Change on Δt = 1 °C
*c*_11_ × 10^10^ Pa	15.1	15.505	4.953 × 10^−4^
*c*_12_ × 10^10^ Pa	7.9	9.172	−7.651 × 10^−4^
*c*_13_ × 10^10^ Pa	8.0	8.711	3.450 × 10^−3^
*c*_33_ × 10^10^ Pa	13.6	13.867	1.653 × 10^−4^
*c*_44_ × 10^10^ Pa	2.9	2.775	−1.461 × 10^−3^
*e*_15_, C/m^2^	15.4	15.470	2.918 × 10^−3^
*e*_31_, C/m^2^	−7.9	−6.427	4.665 × 10^−3^
*e*_33_, C/m^2^	17.7	17.188	−4.885 × 10^−3^
*ε*_11_/*ε*_0_	1610	1439.3	8.758 × 10^−2^
*ε*_33_/*ε*_0_	1280	1220.0	9.334 × 10^−1^

## Data Availability

Not applicable.
